# Modulation of chicken gut microbiota for enhanced productivity and health: A review

**DOI:** 10.14202/vetworld.2024.1073-1083

**Published:** 2024-05-15

**Authors:** Himmatul Khasanah, Dwi E. Kusbianto, Listya Purnamasari, Joseph F. dela Cruz, Desy C. Widianingrum, Seong Gu Hwang

**Affiliations:** 1 Study Program of Animal Husbandry University of Jember, Jember 68121, Indonesia; 2 Applied Molecular and Microbial Biotechnology (AM2B) Research Group, University of Jember, Jawa Timur, 68121, Indonesia; 3 Study Program of Agricultural Science, University of Jember, Jember 68121, Indonesia; 4 School of Animal Life Convergence Science, Hankyong National University, Anseong 17579, Republic of Korea; 5 Department of Basic Veterinary Sciences, College of Veterinary Medicine, University of the Philippines Los Baños, Los Baños-4031, Philippines

**Keywords:** feed additive, metagenome, phytogenic, poultry production, prebiotic, probiotic

## Abstract

Microbiota in the digestive tract has become an interesting topic for researchers in recent years. The profile of chicken digestive tract microbiota and its relationship with health and production efficiency have become basic data for modulating the diversity and abundance of the digestive tract microbiota. This article reviews the techniques used to analyze the diversity, role, and function of the gastrointestinal microbiota and the mechanisms by which they are modulated. The gut microbiota plays an important role in animal production, especially during feed digestion and animal health, because it interacts with the host against pathogens. Feed modulation can be a strategy to modulate gut composition and diversity to increase production efficiency by improving growth conditions.

## Introduction

Chicken is a cost-effective source of animal protein for humans in both developing and developed countries. Chicken farmers and the chicken industry are struggling to maintain good production and performance and achieve economic benefits. Increased production needs to be accompanied by increased production efficiency. In addition, the main challenge faced by the poultry industry is to improve the performance of poultry while ensuring sustainability, cost-effectiveness, and environmentally friendly.

Multiple factors, including genetics, feed, and health management, affect livestock performance and health [1]. In broiler cultivation, feeding incurs the largest production costs, indicating the need to ensure efficiency, directly or indirectly [2]. Therefore, enhancing the broiler’s ability to transform consumed feed into body growth, generally called feed efficiency, is essential for increasing poultry production and ensuring sustainable intensification [3]. Feed efficiency in chickens is associated with gut health [4]. The digestive tract (monogastric tract) of chickens is smaller but faster than that of mammals [5]. The tract has a crop where feed is stored and delivered to the proventriculus (true stomach), and a gizzard where the feed is mechanically digested before entering the small, large, and ceca [6]. This tract denotes the microbiota habitat that affects the chicken digestive system. The gut microbiota plays pivotal roles in essential biological processes, such as physiological aging in humans, methane emission in dairy cows, nutrient digestion, absorption, and metabolism in pigs [7], and health and productivity in chickens [8]. It is related to microbiota functions in the digestive tract, such as supporting digestion and providing nutrients to the host. Research has found interesting interactions between the microbiota and the host [9]. Gut health interaction is defined as the symbiotic equilibrium state between the microbiota and the intestinal tract, meaning that animal health and welfare remain unaltered and are considered important factors [10]. The gut microbiota also affects the health and immune system [11]. The microbiota of chicken digestive tract can also determine human antibiotic resistance and infection [12]. In addition, health of this microbiota is associated with the productivity and efficiency of chicken production. Measures to increase chicken production through feed modification aim to modulate the diversity and abundance of gut microbiota. These measures relate to the use of feed additives, feed supplements, and feed processing. Feed additives, such as probiotics, prebiotics, enzymes, amino acids, and phytobiotics, have been reported to increase livestock production [13, 14].

This study aimed to review the diversity, roles, and functions of microbiota in the digestive tract of broiler chickens. This study also elucidates the influence of the modulation of chicken gut microbiota on production performance and health.

## Techniques for Analyzing the Diversity of Microbiota in Digestive Tract

Gastrointestinal microbiota studies were initially conducted using microorganism cultures in bacterial growth media [15, 16]. However, previous studies by Gong *et al*. [17] and Wei *et al*. [18] have struggled with adjusting the environmental conditions in the digestive tract, such as anaerobic conditions, the presence of microbes that must be cultured simultaneously due to simultaneous interaction with metabolism, and difficulties in culturing some unidentified microbiota. Analysis and identification techniques for these microbiotas begin using genomic fingerprints as a solution for microbiota that would otherwise not be cultured. Genetic fingerprinting uses denaturing gradient gel electrophoresis [19], single-strand conformation polymorphism technique, temporal temperature gradient gel electrophoresis [17], and terminal restriction. Terminal restriction fragment length polymorphism (T-RFLP) [20, 21] has limited sensitivity because it cannot detect taxa abundance >1%. In addition, these techniques are lengthy and costly, making them inefficient in identifying the diversity and abundance of microbiota in the digestive tract [18].

**Figure-1:**
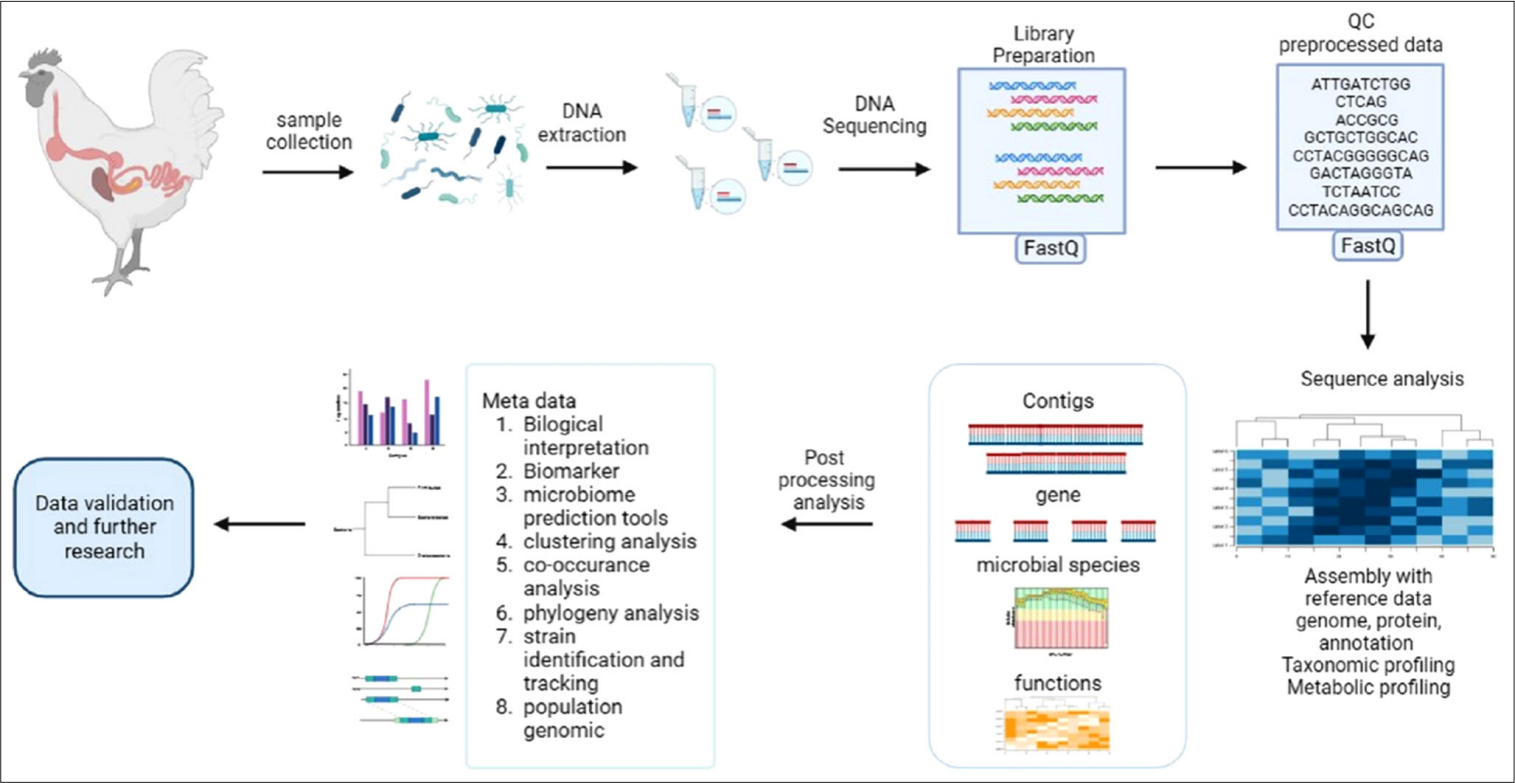
Metagenome pipeline and workflow steps. 1. Design the experiment; 2. Sample collection and preparation; 3. Library preparation (pre-processing and QC); 4. Sequence analysis; 5. post-processing and 6. Validation [Source: Biorender.com].

At present, metagenomics, a technique for analyzing the genome of micro-organisms without culturing, is the most widely used technique. The term “metagenomics” comes from the word “meta,” which refers to a combination of various methods. In contrast, the term “genomics” refers to the holistic analysis of the genetic material of an organism [22]. The analysis focuses on genes with similar sequences, such as ribosomal RNA (rRNA), 16S rRNA for prokaryotes (bacteria and archaebacteria), and 18S rRNA for eukaryotes [22]. Metagenomics can use a high-throughput next-generation sequencing technique to generate data from large-scale analyses and analyze the complexity of environmental communities [23]. An alternative technique for evaluating the abundance and diversity of microbes in an ecological sample, such as soil, digestive tract, feces, plant roots, and fermentation products, is provided. This technique makes it possible to identify uncultured micro-organisms more effectively than culturing media using serial dilution. This technique can result in bias due to discrepancies between the nutrients required by bacteria and those present in the media [24]. Neelakanta and Sultana [25] explained that 16SrRNA analysis has nine hypervariable regions commonly used for polymerase chain reaction primers (Figure-1). The taxonomic estimation accuracy can be generated by comparing 16SrRNA sequences against database sequences. Several primers generally used to analyze the metagenome of chicken digestive tract are displayed in Table-1 [26–35]. Primer selection for sequencing mainly affects the data on alpha-and beta-diversity, taxonomic composition, and predicted functions; V1–V3, V3–V4, and V3–V5 are mostly in consensus with better data [36].

**Table 1:** Hypervariable regions of 16S rRNA generated using gut microbiome metagenome analysis.

Hypervarible region	Forward	Reverse	References
V4–V6	GTGCCAGCMGCNGCGG	GGGTTNCGNTCGTTG	[26]
V1–V3	GAGAGTTTGATYMTGG CTCAG	ACCGCGGCTGCTGGCAC	[27]
V1–V3	DAGAGTTTGATCMTGG CTCAG	TMTTACCGCGGCNGCT GGCAC	[28]
V3	GATCCTACGGGAGGC AGCA	CTTACCGCGGCTGCTGGC	[29]
V4	GTGCCAGCMGCCGCG GTAA	GGACTACHVGGGTWT CTA T	[30]
V3–V4	ACTCCTAC GGGAGGCAGCA	GGACTACHVGGGTWT CTAAT	[31]
V3–V4	′CCTAYGGGRBGCASCAG	GGACTACNNGGGTATCTAAT	[32, 33]
V3–V5	CCTACGGGAGGCAGCAG	CCGTCAATTCMTTTRAGT	[34]
V4–V5	GTGCCAGCMGCCGCG GTAA	CCGTCAATTCMTTTRAG TTT	[35]

## Chicken Digestive Tract and the Characteristics of Gut Microbiota

Like other poultry, chickens have smaller intestines and shorter digestion times than mammals. However, chickens are less efficient at digesting protein than pigs but are more efficient than cattle [37]. The digestive tract of chickens consists of several phases. First, a beak is used to collect food that has a forked tongue. The tongue is located in the back of the beak and is used for drinking and moistening the feed. Second, the esophagus transports food and water. The esophagus contains mucous glands that lubricate the feed and serve as a place where pre-digestion is performed with the aid of the enzyme ptyalin from saliva and amylase from the duodenum and proventriculus. The proventriculus, into which the feed flows slowly, helps to digest the feed using gastric juices (hydrochloric acid and digestive enzymes).

The proventriculus also helps to break down nutrients and creates a bolus that travels into the gizzard. Fourth, the gizzard produces pepsin for proteolytic activity in the proventriculus. In addition, the gizzard functioned as a masticatory organ and smoothed insoluble grains. Fifth, the food enters the small intestine, which is equipped with villi. In addition, protein digestion occurs in the small intestine from intestinal secretions by aminopeptidase, amylase, maltase, and invertase enzymes. In addition, the small intestine absorbs digested nutrients so that these nutrients can be infused into the bloodstream. In addition, the small intestine undergoes peristaltic movements that push undigested feed material into the cecum. Histologically, the small intestine is divided into the following three parts: Duodenum, jejunum, and ileum. The pancreas then secretes amylase, trypsin, lipase, and carboxypeptidase. Next, the liver secretes bile into the duodenum and breaks down fat. At this point, bile is produced in the liver and stored in the gallbladder. Eighth, the cecum ferments undigested food ingredients such as non-starch polysaccharides (NSPS) in the small intestine. The large intestine is emptied every 24 h. The small intestine absorbs the rest of the digested substances (e.g., non-starch polysaccharides), and the large intestine absorbs water. Finally, as an external drain, the cloaca expels feces [38, 39].

The microbiota in the digestive tract varies according to the feed, location, and age of chicken. The diversity of gut microbiota has been reported in the previous studies. Wei *et al*. [18] comprehensively analyzed 16S rRNA sequence data available in public databases. They revealed 3184 quality sequence data collected from GenBank, the Ribosomal Database Project, and the Silva Comprehensive rRNA database. Phylogenetic analysis identified 915 operational taxonomic units (OTUs) with 3% genetic distance. We classified 13 bacterial phyla, dominated by *Firmicutes* (70%), *Bacteroidetes* (12.3%), *Proteobacteria* (9.3%), and cecal bacteria. Sequencing results also revealed 117 genera dominated by *Clostridium*, *Ruminococcus*, *Lactobacillus*, and *Bacteroides*. Furthermore, Shaufi *et al*. [23] reported that several types of similar bacteria are present in the digestive tract, although they function in different locations, namely, in the ileum and cecum. The bacterial phylum *Firmicutes* was dominant in both locations (49–85%). In adult cattle, *Clostridium* XI and *Escherichia coli* are dominant bacteria in the ileum. Only 4% of *Lactobacilli* are present in the ileal tract. In addition, *Enterococcus* (28%), *Escherichia*, *Shigella* (14%), *Clostridium* XI (7%), *Faecalibacterium* (5%), *Alistipes* (5%), and *Bacteroides* (4%) were found in the ileum. However, the bacterial diversity in the cecum was lower. *Bacteroides* (3–22%), *Alistipes* (1–13%), *Faecalibacterium* (3–8%), *Clostridium* XIV *b* (1–3%), *Escherichia*, and *Shigella* (1–5%) are the dominant bacteria in this area. Metagenome studies in chicken cecum have identified five dominant OTUs: *Megamonas*, *Veillonellaceae, Pseudoflvonifractor, Bacteroides*, and *Alistipes* [40]. Oakley *et al*. [41] explained that *Salmonella enterica*, *E. coli*, *Campylobacter*, and *Clostridium perfringens* can interfere with human health. *E. coli* strains that are pathogenic to chickens are derived from the avian pathogenic *E. coli* group, which is associated with intestinal and respiratory infections. The diversity and abundance of microbiota in the digestive tract change in composition as chickens grow.

Multiple factors influence the gastrointestinal microbiota profile between the host and environment [42]. This includes different strains, genetics, and sexes. Twenty-nine different species based on genotype have been reported in 190 microbiota species, and 49 different species have been identified based on sex [26]. Finally, the production system also affects the diversity of microbiota. Litter microbiome and gut microbiota have been reported to be strongly correlated between cecal and litter microbes [43]. The season also influences the diversity of cecal microbes. For example, fewer genera are found in winter than in summer or spring [44]. Farm management also determines the development of gut microbiota, such as vaccination, photoperiod, stocking density, feed, ventilation and airflow, pollutants, ammonia, heat stress, and welfare [45–48].

## The Roles of Microbiota in Feed Digestion

According to Choi *et al*. [49], the microbiota in the digestive tract acts as an engine for nutrient metabolism in the host (chicken). The microbiota in the cecum aids in polysaccharide metabolism because chickens do not have a complete cycle to absorb polysaccharide forms. Polysaccharides produce various short-chain volatile fatty acids (SCFAs), namely, acetate (most dominant), propionate, butylate, valerate, isobutylate, and isovalerate, during digestion [50]. Tang *et al*. [51] reported a correlation between metagenomics and metaproteomics in terms of both protein composition and expression in chickens. Pan and Yu [52] mentioned that the microbiota in the digestive tract supports nitrogen metabolism. The intestinal microbiota also contributes to nitrogen metabolism through the catabolism of uric acid to ammonium from the cloaca to the cecum by modulating metabolism in the cecum, allowing the host to absorb ammonium. This allows chickens to synthesize amino acids [53]. This microbiota digests amino acids because its host lacks enzymes to digest nitrogen [54]. These microbiota streamline nitrogen metabolism and produce healthier and more productive chickens [11]. In addition, gut bacteria provide amino acids [55] and vitamins [56], but most of these proteins and vitamins are lost during excretion. Most intestinal bacteria are found in the cecum, which cannot digest or absorb protein [57]. This process involving microbiota is reciprocal because chickens can also provide nutrients for intestinal bacteria, such as mucin (MUC) produced by calceiform cells in the intestine. These nutrients are important sources of carbon, nitrogen, and energy for commensal and pathogenic bacteria [58]. MUC-degrading bacteria can affect intestinal health because they exert selection pressure against bacteria that cannot adhere to mucosal surfaces [52]. Metagenome studies on chicken microbiota using amplicon V3 gene 16SrRNA have identified several functions of microbiota metabolism, including bacterial protein motility, fructose and mannose metabolism, ribosome biogenesis, amino acid and sugar metabolism, secretory system, flagellar assembly, pantothenate and coenzyme A biosynthesis, bacterial chemotaxis, and vitamin B6 metabolism [23]. The cecum microbiota also plays an important role in the metabolism of non-starch polysaccharides (NSPs) and beta-glycans, which are generally found in poultry feeds, such as wheat and corn. *Actinobacteria*, *Bacetriodia*, *Clostridia*, *Lentisphaerae*, *Negativicutes*, *Proteobacteria*, and unknown bacteria capable of producing NPS-degrading enzymes of crystalline cellulose and more than 200 enzymes, including licheninase, endohemicellulase, oligosaccharide-degrading enzyme, glucanase, arabinoxulanase, and bioassociated cellulase [40]. The microbiota in the cecum can produce SCFAs, such as acetic acid, propionic acid, and butyric acid, by fermenting sugars produced by NSP. SCFA provides several advantages for the host, including providing nutrition for chickens, inhibiting the growth and colonization of pathogens, and increasing mineral absorption [59]. Acetic acid production was confirmed by the discovery of 30 acetate kinase/phosphotransferase sequences. Some genes and enzymes involved in the formation of propionate include methymalonyl decarboxylase owned by *Bacteroidetes*, *Firmicutes*, *Probacteria*, and unknown bacteria such as *Tanerella* spp. from the *Bacteroidales* group and *Megamonas hypemegalae* from *Veillonellaceae*. The cecum microbiota such as *Clostridium leptum* (*Clostridiales*) and *Bacteroides* also produce butyrate [40]. Sergeant *et al*. [40] also explained that microbes in the cecum, including methanogenic bacteria (*Euryarchaeota* group) and sulfate reducers (*Desulfovibrio* spp.), consume hydrogen to produce acetate.

The relationship between feed efficiency and gut microbiota diversity showed that the diversity in the jejunum was not significantly different between chickens with high feed efficiency and those with low feed efficiency. On the other hand, there is a significant difference between these groups of chickens. Different abundance represents target populations that can be modified using prebiotics and probiotics to improve animal growth [60].

## The Roles of Microbiota in Supporting Chicken Immune System

Pathogenic bacteria such as *Salmonella*, *Clostridium*, *Campylobacter*, *Staphylococcus*, and *E. coli* that affect chicken health are also found in the digestive tract [41]. Several microbes pathogenic to humans, such as *Campylobacter*, do not necessarily affect chicken. Oakley *et al*. [41] reported no differences in the microbiota of the digestive tract with or without antibiotics. Stanley *et al*. [11] explained that administration of mannan-oligosaccharide increases the number of *Firmicutes* but does not affect *Bacteroides*. However, it reduces the number of *E. coli* and *Salmonella* in the cecum.

Microbiota plays a pivotal role in modulating the activation and regulation of the immune system of broiler chickens. As part of the innate immune response, the intestinal mucosa is the first defense against infection and a barrier that prevents commensal bacteria from penetrating the intestinal epithelium [61]. In addition, the innate immune system provides direct protection by identifying conserved microbial patterns or other indicators of host cellular damage [62]. The interior surface of the avian gut is covered with a mucous layer comprising glycoprotein MUC secreted by calceiform epithelial cells [63]. The physical and chemical barriers of the chicken gut protect against harmful agents. These obstacles are reinforced by subepithelial immune cells and their interaction. The mucus layers provide the first line of protection, facilitate entrapment, and discard interfering bacteria through the luminal stream. On the other hand, the mucus layers also provide colonization spots and nutrients for the gut microbiome [64]. Goblet cells contribute to the production of extensive glycoprotein MUCs, which form mucus layers. These MUCs can be secreted or bound to membranes. The mucus layers produced during antimicrobial secretion protect the intestinal epithelium. Paneth cells and enterocytes provide host defense peptides. Host defense peptides (HDP) supports a broad spectrum of antimicrobial properties against pathogens, such as bacteria, viruses, fungi, and protozoa [65] and promote inflammation settlement, wound recovery, and adaptive immune response development [66]. Various classes of HDP have been identified, including defensins, cathelicidins, S100 proteins, RNase A superfamily, regenerating islet-derived III (REGIII) C-type lectins, and peptidoglycan-recognition proteins. No α-defensins, 14 β-defensins, 4 cathelicidins, and S100 proteins are encoded in chicken genome [66]. Furthermore, lamina propria plasma cells secrete immunoglobulin A, which plays various roles in maintaining mucosal immunity, including neutralizing micro-organisms or toxigenicity and maintaining commensal microbes and intestinal homeostasis [67]. Some other biomarkers vital for gut health in chickens are serum endotoxin and α1-acid glycoprotein (AGP), gene expression of fatty acid-binding protein 2 (FABP2), fatty acid-binding protein 6 (FABP6), interleukin (IL)-1β, IL-8, transforming growth factor-β4, MUC2, and occludin in the mucosa [68]. Compared to sulfated MUCs, which are common in birds with a low number of bacteria due to strict biosecurity, MUCs with higher levels of sialic acid are found in conventionally reared (i.e., coop-dwelling) chickens. This difference was evident on day 4 after hatching, indicating that the microbiota also regulates mucosal layer formation [69]. The microbiota also regulates the production of antimicrobial peptides on the surface of the intestinal epithelium, thereby facilitating rapid elimination or suppression of bacterial activity.

The microbial community of the digestive tract affects the development of the chicken digestive tract. Investigation of the gut histology and microbiota diversity in conventional and germ-free chickens revealed differences [70]. A less mature small intestine mucosa is found in germ-free chicken, which reduces the density and number of goblet cells, increases sulfated MUC, and decreases MUC2 mRNA expression in the small intestine.

## Modulation of Diversity and Abundance of Chicken Gastrointestinal Microbiota

Modulating gut microbiome composition, known as dysbiosis, is driven by several factors such as diet, diseases, stress, and antibiotics. At present, several methods have been applied to modulate the intestinal microbiome, such as dietary modulation, use of antimicrobials and antibiotics, probiotics, prebiotics, postbiotics, synbiotics [71], phytogenic agents, enzymes, organic acids, and other feed additives. Other methods, such as in ovo probiotic administration from adult microbiota, promote the diversity of chickens and reduce the prevalence of pathogenic bacteria [72]. Newly hatched chicks rely on innate immune responses until the gut microbiome develops. One strategy to boost and stimulate chicken immune development (pre-hatching and post-hatching) is microbial administration [71].

In contrast, adding beneficial substances to strengthen the gut epithelial barrier helps to regulate beneficial microflora populations, and the resulting metabolites can be a promising strategy for regulating microflora. Modulating the diversity and richness of the microbial gut is also associated with enhanced anti-inflammatory gene regulation, promoting animal health [73]. Other feed additives such as β-mannanase can modulate gut histology (increase mucosal thickness, higher villus, and lower crypt depth) and intraepithelial lymphocyte number [74]. β-mannanase can also mitigate the impact of coccidiosis on gut microbiota composition and diversity. These strategies are presented in Table-2 [27, 29, 33, 73, 75–89].

**Table 2:** The chicken gut modulation strategies.

Strategy	Treatment	Broiler performance	Microbial modulation	Identification technique	References
Probiotic supplementation	*Bacillus amyloliquefaciens*- based direct-fed microbials	Increased Body weightgain and improved Feed conversion ratio, increased digestibility, increased villus height and ratio of villus and crypts depth.	Decreased cecal *Escherichia coli* and increased *Lactobacillus* population.	Bacterial culture	[76]
	*Bacillus* *amyloliquefaciens*	Increased average daily gain, serum IgG, and IgA; decreased fecal NH_3_ and H_2_S emissions.	No significant effect on cecal *Lactobacillus* and *Bacillus*; negative effect on cecal *Escherichia coli*.	Bacterial culture	[77]
	*Bacillus* *amyloliquefaciens*	Improved body weight and average daily gain; modified cecal metabolites involved in amino acid and glyceride metabolism.	Predominant microbiota are *Ruminococcaceae, Lachnospiraceae, Enterobacteriaceae, Erysipelotrichaceae, Lactobacillaceae,* and *Rikenellaceae*; increased *Enterobacteriaceae* on day 42; increased relative abundance of *Faecalibacterium* and *Ruminococcus* on day 21; increased *Faecalibacterium* and *Blautia*; and decreased *Ruminococcus* on day 42.	HiSeq high-throughput sequencing analysis of 16S rRNA	[29]
Prebiotic supplementation	Red seaweed supplementation	No significant effect on feed intake, BW, egg production, fecal moisture content, and blood serum profile; increased egg weight, egg yolk weight, villus height, and villus surface area.	Increased abundance of *Bifidobacterium longum* and *Streptococcus salivarius*; reduced prevalence of *Clostridium perfringens*.	Quantitative real-time PCR	[78]
	Bambermycin and sophorolipid	Increased feed conversion; increased expression of interleukin-10, claudin-1, and mucin 2 genes.	Increased *Lactobacillus* and *Streptococcus*; decreased *Streptococcus Gallolyticus, increased Akkermansia Muciniphila.*	V3–V4 regions of 16S rDNA sequencing	[79]
	Galacto- oligosacharide supplementation	Increased body weight, longer villi, and deeper crypts. Upregulated IL-17A and IL-17F and downregulated IL-10. Positive correlations between abundances of the *Lactobacillus* isolates with bird weight.	No significant difference in alpha diversity and community richness in cecum. Increased *Lactobacillus crispatus* and *Lactobacillus johnsonii.*	16S rRNA gene V4 region	[73]
	Inuline + white bran	Increased body weight on days 7, 11, and 35; lower feed conversion ratio; higher villus height in jejunum and ileum; higher acetate concentration in ceca; and higher villus and crypst ratio.	No significantly different effect on gut microbiota.	V1-V3 region of the 16S rDNA sequencing	[27]
Natural antimicrobia and Phytobiotic supplementation	Bacitracin	Increased body weight and FCR	Decreased *Bifidobacterium* while other bacterial groups were affected only at certain times.	16S rRNA High-throughput sequencing	[80]
	Garlic derivated propyl propane thiosulfonat	Increased digestibility	Modulated microbial composition in crop, ileum, and cecum	Real-time PCR	[81]
	Tannin suplementation	Increased body weight and FCR	Decrease in *Bacteroides* genus and increased order *Clostridiales*, mainly *Ruminococcaceae* and *Lachnospiraceae.*	16S rRNA High-throughput sequencing	[80]
	Bioactive phenolic extracts from blueberry (*Vaccinium corymbosum*) and blackberry (*Rubus fruticosus*) pomaces	Increased body weight, modulated relative abundance of genes involved in energy and carbohydrate metabolism	Increased *Firmicutes* to *Bacteroidetes* ratios.	Metagenome analysis	[82]
	Plant extract	Increased average body weight, lower feed to meat ratio, several metabolic pathways including cellular processes and signaling, metabolism of cofactors and vitamins, and infectious diseases, cecal metabolism pathway: xenobiotics biodegradation metabolism and enzyme families Inceased functions associated with the carbohydrate metabolism and the digestive system	Decreased *Lactobacillus in cecal, aecalibacterium* and increased unclassified *Rikenellaceae*.	V3–V4 region of the 16S rRNA amplicon pyrosequencing.	[83]
	*Phyllantus urinari*	-	Increased OTU number, higher *Lactobacillus.*	16SrRNA amplicon sequencing	[33]
	Allium-based phytobiotics	Increase egg number, FCR, not differences on egg weight, albumin height, haugh units, eggshell strength, and eggshell thickness. Decrease egg yolk color	Presence of *Firmicutes* and *Bacteroidetes* but reducing *Proteobacteria* and *Actinobacteria* phyla.		
Other feed additive	Adding emulsifier and xylanase in wheat-based diets with beef tallow	Reduce digesta viscosity, several bacterial enzyme activities (galactosidase and glucosidase in ileum, galactosidase, and beta glucuronidase in cecum), increase valerate in ileum, butyrate and total Short Chain Fatty Acid (SCFA) in cecum	Reduced ileum microbiota activity and enhanced cecum microbiota activity, reduced *Clostridium* spp.	Several bacterial 16S rRNA amplification	[84]
	L-theanine supplementation	Decreased mRNA expression of Toll-Like Receptors 2 ( TLR -2), TLR-4, Tumor Necrosis Factor alpha (TNF-a), Interferon gamma (IFN-g), and Interleukin 2 (IL-2), increase mucosal protein ZO-1, occuludin.	Increased population of *Lactobacillus* in ileum and jejunum, lower microbiome diversity of the jejunum. Increased beneficial bacteria (*Lactobacillus)* and decreased *Clostridium*	V3–V4 region of the 16S rRNA Miseq Sequencing	[85]
	β-mannanase	Alleviating effect of coccidiosis in intestine	Dominated bacteria were family of *Ruminococcaceae* and *Lachnospiraceae* and genera of *Faecalibacterium* and *Bacteroides*, and the order *Clostridiales. Beneficial bacteria increased including Lactobacillus, Ruminococcaceae*, and *Akkermansia. Reduced Bacteroides.*	V3–V4 region of the 16S rRNA gene Miseq sequencing	[75]
	Phytase combined with Ca and dP level in diet	Reduce Ca and dP in the diet reduced total SCFA, acetic acid and DL-lactate in ileum. Phytase increased SCFA, acetic acid and DL-lactate in ileum in the insufficient Ca and dP group	Increased ratios of *Lactobacillus* spp. and *Enterococcus* spp.	Fluorescence *in situ* hybridization	[86]
	MCFA and Organic acid	increased duedenum villi and decreased crypth depth (organic acid showed better villus histology) Increased acetic, propionic, butyric, isobutyric, valeric and isovaleric acids in cecum Increased body weight and carcass parameter, lower mortality and increased meat’s yellowness and water holding capacity.	Predominant genera were *Clostridium, Escherichia, Enterococcus*, and *Natranaerovirg*. *Decreased Blautia, Pappilibacter*, and *Bacteroides* genera. Increased probiotic species (*Lactobacillus* and *Bifidobacterium*) in cecum.	Metagenome analysis in cecum	[87]
Feed processing	Fermented soybean meal	No difference in ADG between fermented and unfermented soybean meal, Fermented suplementation increased serum IgA, IgG, and IgM	Increased genera *Lachnospiraceae, Lachnoclostridium, Gastranaeroph*ilales, and *Lactobacillus,* decreased abundance of *Escherichia,-Shigella* and *Clostridiales* in cecum.	16s rDNA sequencing	[88]
	Defatted olive oil by-product as feed	Increase villus height, crypth depth, digestibility, body weight on 35 and 42 days	Not significant in bacterial taxonomy variability and abundance at genus level.	V3–V4 region of the 16S rRNA sequencing	[89]

FCR=Feed conversion ratio, ADG=Average daily gain, IL=Interleukin, Ig=Immunoglobulin, PCR=Polymerase chain reaction, TNF-α=Tumor necrosis factor alpha, IFN-γ=Interferon gamma

## Conclusion

Sequencing techniques are widely used for microbiota identification and functional gene pathways. Effective production strategies include optimizing the gut microbiota to achieve better production and health status and modulating the gut microbiota, such as providing feed additives in combination with diet. The ban on antibiotic growth promoters provides a new opportunity to explore and apply feed additives such as probiotics, prebiotics, phytogens, organic acids, enzymes, and other feed additives in the poultry industry. Feed processing can also improve chicken production, health, and microbiota. These treatments were successful in modulating gut microbiota. Gut bacteria produce various metabolites that may benefit or harm the host. The role of microbiota in the physiological, developmental, nutritional, and immunological processes of the host has a beneficial effect on the gut health, performance, and well-being of poultry in a range of ways. Beneficial bacteria can protect the host from pathogenic bacteria through various competitive mechanisms. These bacteria contribute to the development of the intestinal immune system. Microbiota can significantly hinder growth performance due to the enormous loss of proteins and high metabolic energy expenditure. It may also have a negative impact on vitamin nutrition. Therefore, modulation of gut microbiota is very important in the post-antibiotic era. As reviewed in this paper, alternatives to antibiotics, such as probiotics, prebiotics, organic acids, and exogenous enzymes, tend to modulate gut microbiota. After a thorough understanding of the role of these dietary supplements in the overall performance of poultry, the next step would be to identify alternative sources (plants, animals, or other origins) rich in these supplements. In addition, studies focusing on the combination of these feed additives for their synergistic and agonistic effects may contribute to filling the gap in information on their combined effects.

## Authors’ Contributions

HK and DCW: Conceptualization, literature review, and drafted the manuscript. DEK, JFC, LP, and HSG: Performed the manuscript writing and literature review. All authors have read, reviewed, and approved the final manuscript.
